# A Long Short-Term Memory Network for Plasma Diagnosis from Langmuir Probe Data

**DOI:** 10.3390/s22114281

**Published:** 2022-06-04

**Authors:** Jin Wang, Wenzhu Ji, Qingfu Du, Zanyang Xing, Xinyao Xie, Qinghe Zhang

**Affiliations:** 1 Institute of Space Sciences, Shandong University, Weihai 264209, China; jinwang@mail.sdu.edu.cn (J.W.); xingzanyang@sdu.edu.cn (Z.X.); messi@mail.sdu.edu.cn (X.X.); 2 School of Mechanical, Electrical & Information Engineering, Shandong University, Weihai 264209, China; 202037483@mail.sdu.edu.cn (W.J.); dqf@sdu.edu.cn (Q.D.)

**Keywords:** LSTM, machine learning, Langmuir probe, plasma diagnosis

## Abstract

Electrostatic probe diagnosis is the main method of plasma diagnosis. However, the traditional diagnosis theory is affected by many factors, and it is difficult to obtain accurate diagnosis results. In this study, a long short-term memory (LSTM) approach is used for plasma probe diagnosis to derive electron density (*N_e_*) and temperature (*T_e_*) more accurately and quickly. The LSTM network uses the data collected by Langmuir probes as input to eliminate the influence of the discharge device on the diagnosis that can be applied to a variety of discharge environments and even space ionospheric diagnosis. In the high-vacuum gas discharge environment, the Langmuir probe is used to obtain current–voltage (I–V) characteristic curves under different *N_e_* and *T_e_*. A part of the data input network is selected for training, the other part of the data is used as the test set to test the network, and the parameters are adjusted to make the network obtain better prediction results. Two indexes, namely, mean squared error (MSE) and mean absolute percentage error (MAPE), are evaluated to calculate the prediction accuracy. The results show that using LSTM to diagnose plasma can reduce the impact of probe surface contamination on the traditional diagnosis methods and can accurately diagnose the underdense plasma. In addition, compared with *T_e_*, the *N_e_* diagnosis result output by LSTM is more accurate.

## 1. Introduction

Plasma is a complex thermodynamic system composed of electrons, ions, and neutral particles, which widely exists in cosmic space. Plasma is a conductive fluid as a whole, showing electrical neutrality macroscopically, but under the action of an electromagnetic field, energy transmission can occur. The measurement of the plasma state has always been the focus of researchers. The state of plasma can be characterized by electron density (*N_e_*), electron temperature (*T_e_*), plasma space potential (*V_p_*), and other parameters, among which the most crucial are *N_e_* and *T_e_*. *N_e_* describes the number of electrons per unit volume, while *T_e_* describes the kinetic energy possessed by electrons. Under thermal equilibrium conditions, *T_e_* is equal to the ion temperature (*T_i_*). Most diagnostic methods for plasma are aimed at obtaining *N_e_* and *T_e_*. The diagnosis methods of plasma are divided into telemetry diagnosis and in situ diagnosis. Telemetry diagnosis includes microwave diagnosis and spectral diagnosis. Langmuir probe diagnosis is the most common in situ diagnosis technology, which has been widely used in laboratory and space plasma detection [1,2,3,4,5,6,7]. Compared with telemetry diagnosis, the Langmuir probe can obtain more reliable and accurate diagnosis results.

However, the traditional diagnostic method of Langmuir probes highly depends on the acquisition of the current–voltage (I–V) characteristic curve. The degree of deviation of the collected I–V characteristic curve from the actual value directly affects the reliability of the diagnosed plasma parameters. The shape of the I–V characteristic curve is affected by many factors, such as the contaminated layer on the probe surface, the sheath and the Debye length of plasma, and even the driving circuit, which makes serious errors in the diagnosis results. In addition, the excessive “human factors” in the traditional diagnosis process also increase the randomness of the diagnosis results.

Since 1938, when using Langmuir probes to diagnose plasma [8,9], many researchers have found the contaminated layer on the probe surface, which is mainly manifested in the hysteresis of the I–V characteristic curve [10]. Two different distorted I–V curves could be obtained by a Langmuir probe, when the applied voltage was swept upward and then downward [2,11,12,13,14]. In order to eliminate the influence of the contaminated layer on data, Oyama [15] applied a glass-sealed Langmuir probe to ionosphere exploration. The reason for abandoning the traditional Langmuir probe is that it easily adsorbs water molecules, nitrogen molecules, and oxygen molecules to form a contaminated layer on the surface of the probe. Before sounding rockets or satellites are launched, Langmuir probes that have been exposed to the atmosphere are easy to be contaminated. Subsequently, in order to avoid the influence of contaminants on the diagnostic results of Langmuir probes, Amatucci et al. [16] invented a spherical Langmuir probe that can remove surface contaminants by internal heating; however, this structure cannot be applied to the most widely used cylindrical probe now. Szuszczewicz and Holmes [17] invented a pulsed Langmuir probe (PLP), which employs a discontinuous modulated sweep of pulses following a sawtooth envelope. They believe that this approach can obtain more accurate plasma parameters than heating or ion bombardment. These new Langmuir probes can reduce the contaminated layer’s influence to a certain extent. However, the structure of restraining contamination increases the complexity of the probe and makes the design of the probe or driving circuit more complex. On the other hand, for the data collected by the contaminated Langmuir probe, Jiang et al. [10] proposed a new iterative algorithm by using a method of establishing an equivalent circuit, but the operation is relatively tedious, and it is difficult to expand the application due to the influence of plasma characteristics.

In the plasma field, researchers have begun to use neural networks to realize the intelligent machine diagnosis of plasma. Kawaguchi et al. [18] utilized machine learning to solve the Boltzmann equation of electrons to obtain an electron distribution function (EVDF) in weakly ionized plasmas. Compared with plasma diagnosis, this network tends to solve a mathematical problem. Churchill et al. [19] utilized convolutional neural networks for tokamak disruption prediction, which is a popular problem in tokamak devices. In the Tokamak, too, Guo et al. [20] realized a long short-term memory (LSTM) model on a large disruption warning database to predict the disruption. To be exact, these two networks are aimed at disruption prediction rather than plasma diagnosis, although the Tokamak device is an application in the field of plasma. In the diagnosis of dusty plasma, Ding et al. [21,22] used the multilayer perceptron (MLP), took the air pressure and voltage or air pressure and current of the discharge device as the input of the perceptron, and trained the network to predict *N_e_* or *T_e_*. However, the network trained by the above method depends on the device’s characteristics and is difficult to apply to other discharge devices or aerospace environments. There are many ways of gas discharge that can produce plasma. What we want to achieve is a more universal method to realize plasma diagnosis. Thus, it is necessary to diagnose the plasma without the parameters of the discharge device, which requires the data collected by Langmuir probes to construct the network. This method can be applied to space plasma diagnosis in the future.

## 2. Traditional Diagnostic Theory and Problems

A Langmuir probe is a small electrode inserted into a plasma. When a scan voltage is applied to it, with the change in voltage, the electrode will absorb electrons or ions, causing current flow and forming an I–V curve [23].

**Figure 1:**
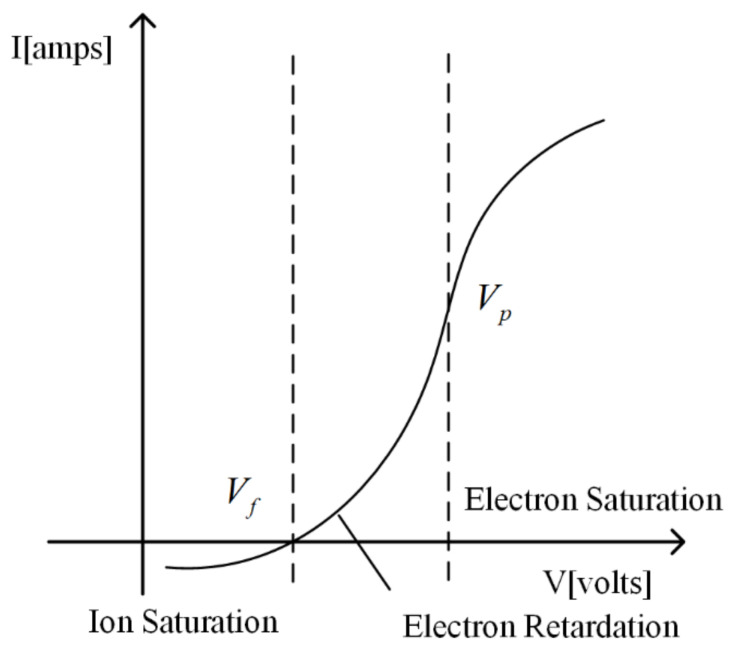
A representative I–V characteristic curve.

In a nondrifting, collisionless, and nonmagnetized plasma, the representative I–V characteristic curve of cylindrical probes is shown in Figure 1. The curve is divided into three regions: ion saturation, electron retardation, and electron saturation. The dividing points are floating potential *V_f_* and plasma potential *V_p_*.

According to orbital-motion-limited (OML) theory [8], the electron current (*I_e_*), ion current (*I_i_*), and Langmuir probe current (*I_LP_*) collected by cylindrical, planar, and spherical probes are as follows:

(1) $$I_{e}=\frac{2}{\sqrt{\pi }}N_{e}Ae\sqrt{\frac{k_{B}T_{e}}{2\pi m_{e}}}\exp(\frac{eV}{k_{B}T_{e}}),\;\;V=V_{B}-V_{p}<0;$$

(2) $$I_{e}=\frac{2}{\sqrt{\pi }}N_{e}Ae\sqrt{\frac{k_{B}T_{e}}{2\pi m_{e}}}{\left(1+\frac{eV}{k_{B}T_{e}}\right)}^{\beta },\;\;V=V_{B}-V_{p}>0;$$

(3) $$I_{i}=\frac{2}{\sqrt{\pi }}N_{i}Ae\sqrt{\frac{k_{B}T_{i}}{2\pi m_{i}}}\exp(\frac{eV}{k_{B}T_{i}}),\;\;V=V_{B}-V_{p}>0;$$

(4) $$I_{i}=\frac{2}{\sqrt{\pi }}N_{i}Ae\sqrt{\frac{k_{B}T_{i}}{2\pi m_{i}}}{\left(1+\frac{eV}{k_{B}T_{i}}\right)}^{\beta },\;\;V=V_{B}-V_{p}<0;$$

(5) $$I_{LP}=I_{e}+I_{i};$$

where *N_i_* is the ion density, *A* is the surface area of the probe, *e* is the electron charge, *k_B_* is Boltzmann’s constant, *T_i_* is the ion temperature, *m_e_* is the electron mass, *m_i_* is the ion mass, and *V_B_* is the voltage applied by the probe. When the probe is planar, cylindrical, or spherical, the corresponding β values are 0, 0.5, and 1.

The critical algorithm in Langmuir probe data processing is the fitting and inversion calculation of the I–V characteristic curve. This method includes the determination of *V_f_* and *V_p_*, obtains the ion saturation current and modifies *I_e_*, the logarithmic fitting of the electron retardation curve to derive *T_e_*, and obtains *N_e_* according to the value of *I_e_* at *V_p_*. The detailed process is as follows:

1. Determine *V_f_* and *V_p_*. *V_f_* is the point where the current of the I–V characteristic curve is 0. At this point, *I_e_* is the same as *I_i_*, and the direction is opposite. *V_p_* is the potential of the plasma relative to the environment, which is the inflexion point of the I–V characteristic curve, that is, the dividing point between the electron retardation and the electron saturation region.
2. Obtain saturated ion current at $V_{B}<V_{f}-4\frac{k_{B}T_{e}}{e}$. Theoretically, the impact of *I_e_* on *I_LP_* at this point is less than 1%, which can be ignored. Then, the *I_e_* is derived by subtracting the ion saturation current from *I_LP_*.
3. *T_e_* is derived by logarithmic fitting of a section in the electron retardation curve. It can be seen from Equation (1) that there is an exponential relationship between *I_e_* and *V_B_* in the electron retardation region. Find the logarithm of Equation (1) and simplify it to obtain the following equation.
  (6) $$\ln(I_{e})=\frac{e}{k_{B}T_{e}}V+\ln(CI_{e0})$$
  where $C=\frac{2}{\sqrt{\pi }}$, $I_{e0}=N_{e}Ae\sqrt{\frac{k_{B}T_{e}}{2\pi m_{e}}}$. We can obtain *T_e_* from the slope of Equation (6).
4. Derive *N_e_* from Equation (7). When *V_B_* = *V_p_*, *I_e_* = *I_e0_*, bring in the calculated *T_e_* and obtain *N_e_*.

(7) $$I_{e}{}_{0}=\frac{2}{\sqrt{\pi }}N_{e}Ae\sqrt{\frac{k_{B}T_{e}}{2\pi m_{e}}}$$

Unlike the representative I–V characteristic curve, the influence of the contaminated layer on the probe surface and the edge effect or sheath of the low-density plasma will make the collected I–V characteristic curve deviate from the standard form, resulting in the failure of the traditional diagnosis method. The influence of the two factors on the diagnosis method is discussed below.

### 2.1. Contaminated Layer on the Probe Surface

**Figure 2:**
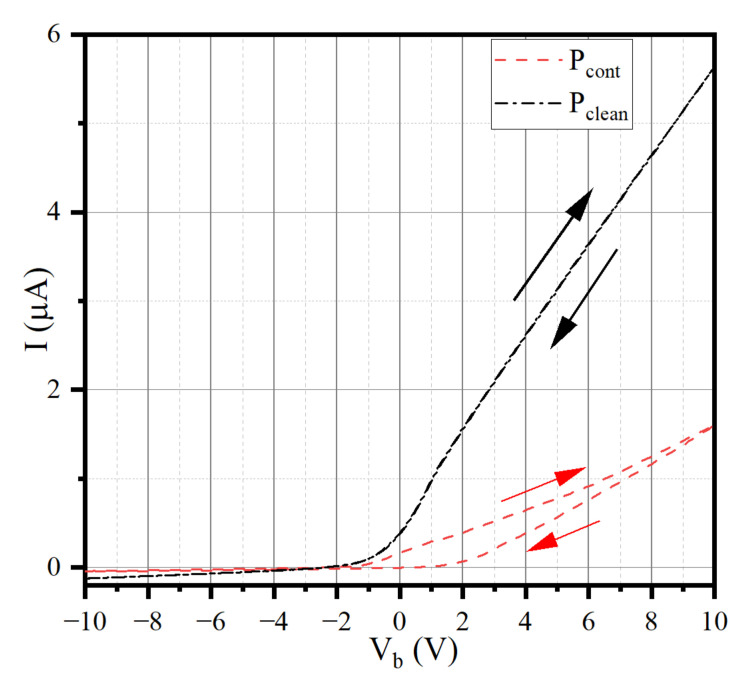
Comparison of I-V characteristic curves collected by contaminated probe and clean probe.

Previous studies have shown that the contaminated layer will change the uniform potential distribution on the probe surface and skew the collected data, resulting in the wrong plasma parameters [2,10,11,16]. In addition, the adsorption process of neutral gas molecules to the probe in the atmosphere can be completed in as short as 1 s. Therefore, the data collected by the probe are affected to varying degrees by the contaminated layer. When the I–V characteristic curve is collected by the probe with serious contamination, the upward curve and downward curve cannot coincide, as shown by the red line in Figure 2.

Figure 2 shows the comparison of the curves collected by the clean probe (P_clean_) and the contaminated probe (P_cont_) in the same ambient plasma. P_cont_ is a probe that has been exposed to the atmosphere. P_clean_ is a probe that has been bombarded by ions with a voltage of −200 V.

This set of data comes from our previous experiments and is collected by the B2912A precision Source/Measure Unit (SMU) of KEYSIGHT [24]. The source and measurement resolution of this SMU can be as low as 10 fA and 100 nV [24]. All experimental data in this study come from this SMU.

In fact, because the experimental device cannot always maintain a high vacuum, there are inevitably gas molecules and water molecules in the cabin. Therefore, before each experiment, we need to clean the probe; otherwise, the curve is as shown in Figure 2.

We use the clean data and contaminated data shown in Figure 2 for plasma diagnosis, and the comparison of plasma parameters is shown in Table 1.

The upward and downward curve of the P_clean_ basically coincide, and the calculated *N_e_* and *T_e_* errors are within 2%. However, the errors of *N_e_* and *T_e_* obtained from the upward and downward curves of P_cont_ are 11% and 80%, respectively. More importantly, in the same ambient plasma, the *N_e_* obtained by the clean probe and contaminated probe can be as much as two times different, and the *T_e_* error is more than 15%, too.

When using the contaminated curve shown in Figure 2 for plasma diagnosis, it will face the phenomenon that the inflexion points of the upward curve and the downward curve are inconsistent. This situation is caused by the contaminants of the probe rather than the change in the plasma, which makes the plasma parameters obtained by diagnosis inevitably have errors.

### 2.2. Underdense Plasma Diagnosis

Due to the relatively small surface area of the cylindrical Langmuir probe, when the density of the ambient plasma is low, the probe collects fewer electrons and ions, and the probe current is very weak, resulting in a low signal-to-noise ratio (SNR) of the collected signal, which increases the difficulty of the data fitting process.

**Figure 3:**
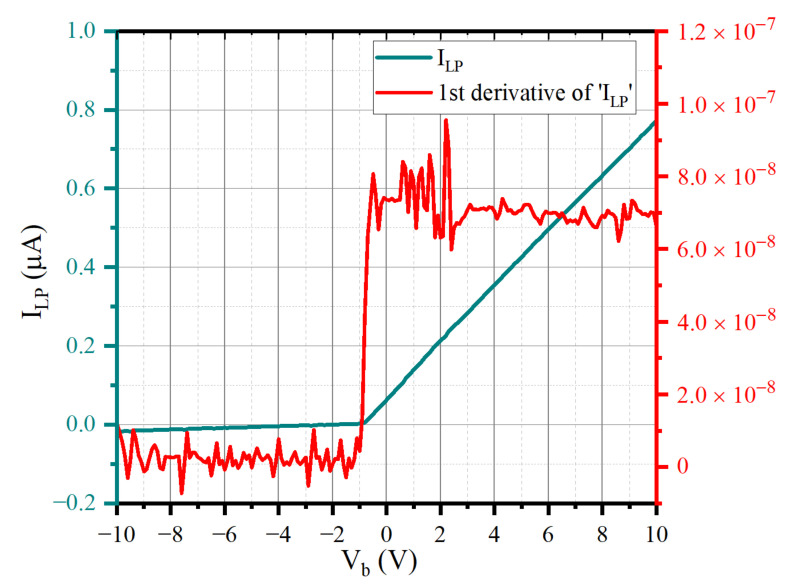
The I–V characteristic curve without obvious saturation region.

In addition, the plasma with lower density has a larger Debye length and sheath width. As the voltage increases, the electron sheath around the probe gradually expands. The OML theory assumes that all electrons entering the probe sheath will be absorbed by the probe [8]. Due to the growth of the sheath, more electrons enter the sheath and the current collected by the probe increases, and the I–V characteristic curve has no obvious saturation point; that is, it is difficult to obtain the correct inflexion point from d*I_LP_*/d*V_B_* data, as shown in Figure 3. This I–V characteristic curve comes from the plasma of *N_e_* = 2.38 × 10^11^ m^−3^ and *T_e_* = 0.3 eV.

For this I–V characteristic curve, the computer program cannot be used for automatic diagnosis, but experienced researchers are required to manually select the inflexion point and the range of the electron retardation region to carry out the next diagnosis program. However, this selection process has great randomness, resulting in significant errors in the diagnosis results. All of these can lead to distortions of the probe’s I–V characteristic due to spatial inhomogeneities in the probe’s contact potential with the plasma and in the collected current.

Aiming at the problems exposed by traditional diagnosis methods in plasma diagnosis, we plan to implement the machine learning method to solve them.

In this paper, we first select a suitable network structure, then obtain a large amount of data for model training, and preliminarily adjust the parameters of the model. Then, the model is trained iteratively, and the accuracy of the network prediction results is continuously tested. In order to make the network use the contaminated data to obtain relatively accurate results, it is also necessary to optimize the network parameters. Eventually, the network should have the following characteristics:

1. *N_e_* and *T_e_* can be obtained by using the relatively rough I–V characteristic curve;
2. Plasma diagnosis can be realized by using the data collected by a certain degree of contaminated Langmuir probe;
3. It can realize low temperature and low-density plasma diagnosis.

## 3. Machine Learning

### 3.1. Principle of LSTM

**Figure 4:**
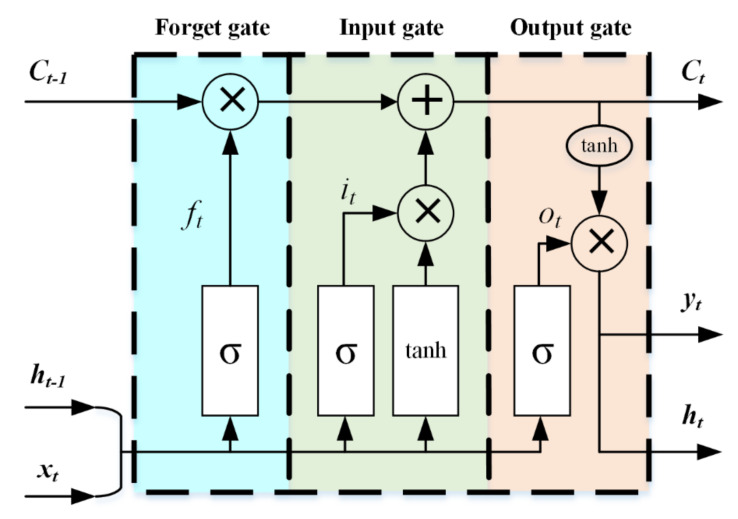
LSTM cell structure.

It is a standard regression problem to predict *N_e_* and *T_e_* using I–V data collected by Langmuir probes. Many neural networks can achieve this function. Ding et al. [21,22] built an MLP network and predicted *N_e_* or *T_e_* by using the state parameters of the discharge device. MLP is a kind of lightweight neural network that is easy to use. However, considering that the data collected by the probe may be affected by the coupling of multiple factors, for example, for the data affected by the contaminated layer on the probe’ surface to varying degrees, the network should have the memory ability to correct the output value well. At this time, MLP is not competent for such tasks, because of its internal structure (one-way propagation from the input layer to multiple hidden layers to the output layer). LSTM is a kind of recurrent neural network (RNN) with a special structure. Compared with the traditional RNN, the hidden layer unit in LSTM is a linear self-cyclic memory block, which contains three gate structures, which allows the gradient to pass through a long sequence, solves the vanishing gradient problem, and overcomes the shortcomings of the RNN model [25]. The basic structural unit of the LSTM network is shown in Figure 4.

A cell of the LSTM network is mainly composed of three parts: forget gate, input gate, and output gate. The input data of LSTM are *x_t_*, *h_t − 1_*, and *C_t − 1_*, and the output data are *h_t_*, *y_t_* and *C_t_*, where *h_t_* = *y_t_*. The main function of the forget gate is to filter the data of the previous state, which determines how much old state information is retained. The calculation equation is shown as follows:

(8) $$I_{e}{}_{0}=\frac{2}{\sqrt{\pi }}N_{e}Ae\sqrt{\frac{k_{B}T_{e}}{2\pi m_{e}}}$$

where *f_t_* denotes the forgetting threshold at time *t*, σ is the sigmoid activation function, *W_f_* is the weight, *h_t − 1_* is the output value at time *t − 1*, *x_t_* is the input value, and *b_f_* is the bias term.

The input gate is used to record the information to be saved in the current state. The input gate consists of two parts: the sigmoid layer to update the value and the tanh layer for generating a new state value *C_t_*. The output of the two layers is as follows:

(9) $$i_{t}=\sigma (W_{i}*[h_{t-1},x_{t}]+b_{i})$$

(10) $$C_{t}{}^{\prime }=\tanh(W_{c}*[h_{t-1},x_{t}]+b_{c})$$

where *i_t_* is the input threshold at time *t*, *W_i_* and *W_c_* are the weights, and *b_i_* and *b_c_* are bias terms. To update the state at time *t*, the expression is as follows:

(11) $$C_{t}=f_{t}*C_{t-1}+i_{t}*C_{t}{}^{\prime }$$

The main function of the output gate is to calculate the output value and make the corresponding prediction results. The equation is described as follows:

(12) $$o_{t}=\sigma (W_{h}*[h_{t-1},x_{t}]+b_{h})$$

(13) $$y_{t}=h_{t}=o_{t}*\tanh(C_{t})$$

where *o_t_* is the output threshold at time *t*, *h_t_* is the output value of the cell at time *t*, *W_h_* is the weight, and *b_h_* is the bias term.

**Figure 5:**
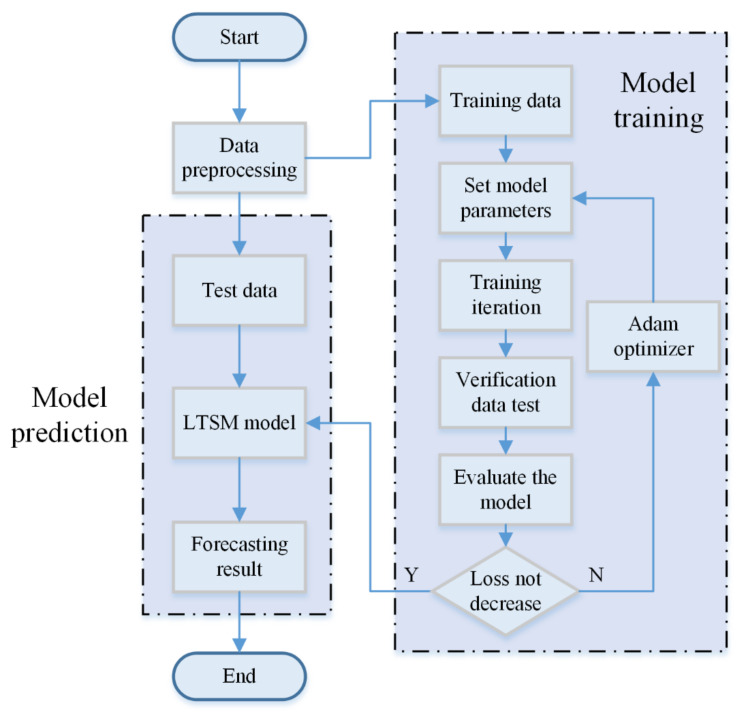
The full flowchart of the LSTM model.

In this paper, LSTM is selected to predict *N_e_* and *T_e_*. In order to better mine the deep-seated internal relationship of the data, a two-layer LSTM network is used, but it is easy for this to cause the over-fitting. To avoid over-fitting, a random deactivation layer is added after each layer of the LSTM network. Finally, the full connection layer is added to the network, and the linear activation function is adopted, making the network output consistent with the label value. The output value of the network is compared with the actual value, and the Adam optimizer is used to update the parameters. The full flowchart of the LSTM model is illustrated in Figure 5.

### 3.2. Evaluation Indicators

In the process of model training, the accuracy is measured by the loss function. The loss function in this model adopts the mean square error (MSE) loss function, which is often used in the regression prediction model to calculate the loss between the predicted value and the actual value. The MSE loss can be calculated as follows:

(14) $$Loss=\frac{1}{n}\sum_{i=1}^{n}{\left(y_{i}{}^{\prime }-y_{i}\right)}^{2}$$

where *y_i_^’^* is the predicted value, *y_i_* is the actual value, and *n* is the number of samples.

After the model training, in order to better evaluate the performance of the model, this paper mainly uses the mean absolute percentage error (MAPE) as the evaluation index, which can be written as follows:

(15) $$\mathrm{MAPE}=\frac{100\%}{n}\sum_{i=1}^{n}\left|\frac{y_{i}{}^{\prime }-y_{i}}{y_{i}}\right|$$

To more intuitively reflect the prediction effect of the validation set during model training, the average accuracy *Ac* of a batch of data can be calculated based on MAPE. The *Acc* can be expressed as:

(16) Acc=1−MAPE

## 4. Experimental Setup and Results

The experimental preparation, data acquisition, and analysis of prediction results are described in this section.

### 4.1. Experiment Setup and Steps

**Figure 6:**
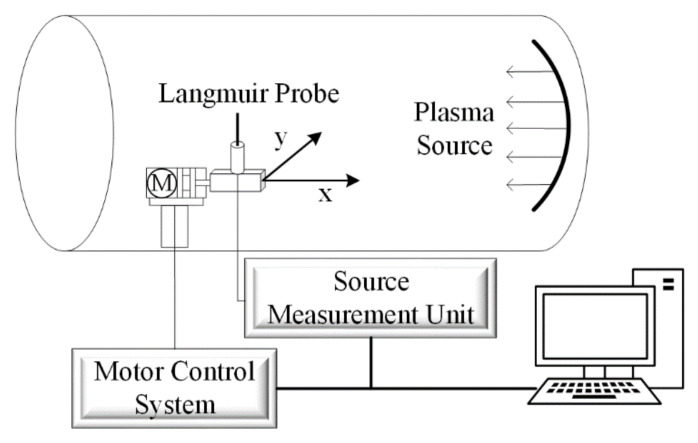
Experimental setup.

The experiment was carried out in a plasma vacuum chamber. The vacuum chamber is a stainless-steel container with a length of 1 m and a diameter of 0.8 m, which can maintain a vacuum of 10^−5^ Pa. The plasma source adopts the DC glow discharge. During discharge, argon is charged to form an argon plasma environment with a gradient density distribution in the cabin. The experimental setup is shown in Figure 6.

In order to obtain the probe acquisition data of the large-scale continuous distribution of plasma density, the Langmuir probe is driven by the two-dimensional motor platform installed in the cabin for omnidirectional acquisition. The motor control system controls the probe to move in the X or Y direction, and the trigger source meter unit collects an I–V characteristic curve every 10 mm forward.

Under the same discharge environment (the same filament current and pressure), the plasma density in the cabin is roughly maintained at the same order of magnitude, but it shows a different density distribution with different distances from the plasma source. When it is necessary to greatly adjust the plasma density in the cabin, it is completed by adjusting the current of the discharge filament.

In order to verify that the neural network can reduce the impact of probe surface contamination on the diagnosis results to a certain extent, without changing the discharge environment (pressure, filament current, etc.), we successively use the probe with the contaminated layer (P_cont_) and clean probe (P_clean_) to collect the I–V characteristic curve at the same position and use the traditional diagnosis method and LSTM network to diagnose the two groups of data to compare the diagnosis results. The specific steps of the comparative experiment are as follows:

1. Expose two identical materials and specifications of the Langmuir probe (P_cont_ and P_clean_) to the humid atmosphere for more than 24 h;
2. Install P_cont_ and P_clean_ on the two-dimensional platform in the vacuum chamber and mark the distance between them and the central position;
3. Heat the filament and charge argon to make the discharge process reach a steady state. Apply −200 V to P_clean_ for ten minutes, and remove the contaminated layer on the probe surface by heating and attracting electrons to bombard the probe surface;
4. Control the two-dimensional platform to move the two probes to the central position to collect the I–V characteristic curve, and ensure that the time interval between the two probe curves is within 1 min;
5. The two groups of collected data are diagnosed and analyzed by the traditional diagnosis method and LSTM network, respectively, to compare the results.

### 4.2. Data Preprocessing

In order to obtain the data of the large distribution range of *N_e_* and *T_e_*, the Langmuir probe is driven by the two-dimensional motor platform to scan in the cabin under the discharge of filament currents 70 A, 80 A, and 85 A, respectively. Under each discharge condition, 2000 groups of I–V characteristic curves are collected. The scan voltage range of each group of curves is −10 to 10 V, the sampling interval is 0.1 V, and a group of data is the current value collected by the probe corresponding to 201 voltages. The whole set of data (201 current values) is used for the traditional method diagnosis. However, only 21 values (interval 1 V) are input into the LSTM network for training or prediction. Note that only the current value is fed into the network training, as the voltage value is fixed. When the probe runs near the bulkhead of the vacuum chamber, it will enter the sheath, resulting in the distortion of the data collected by the probe. Therefore, the dataset should be screened. The total number of data sets available after filtering is 5186 groups.

In addition, due to the large dimension difference of each dimension of data, to eliminate the difference in parameter dimension, data are normalized. The method adopted is max-min normalization, and each group of data is linearly scaled to [0,1], as shown in Equation (17).

(17) $$x^{\prime }=\frac{x-\min(x)}{\max(x)-\min(x)}$$

where *x’* is the normalized value, and max(*x*) or min(*x*) is the maximum or minimum of the dimension where the data are located, respectively.

The dataset is divided into three parts: training set, verification set, and test set according to the ratio of 3:1:1. The training set and verification set are used to train and optimize the model to prevent overfitting, and the test set is used to test the generalization ability of the model.

### 4.3. Results

#### 4.3.1. Network Parameter Setting

There are many adjustable parameters in a neural network, such as the number of neurons in each layer and the learning rate (η). By adjusting these parameters, the prediction accuracy of the model can be effectively improved. In this paper, *N_e_* is mainly used to adjust the parameters and seek the optimal structure.

**Figure 7:**
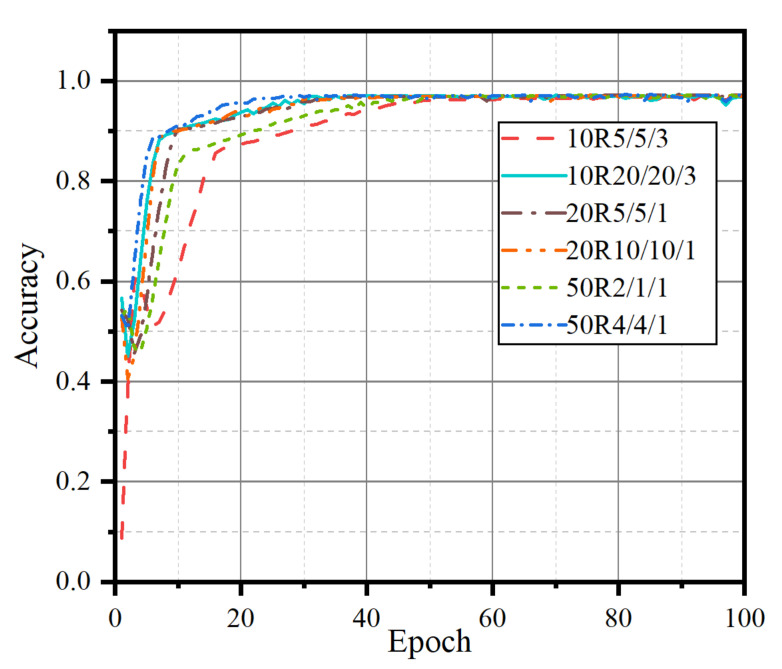
Comparison results of different structures.

The number and proportion of neuron nodes in each layer and the full connection layer of the LSTM network determine the structure of the network and the prediction results. To determine the optimal number and proportion of nodes in each layer, we extract 1000 groups of data in proportion under the data of filament currents 70 A, 80 A, and 85 A for training iteration and we test the accuracy of network prediction of *N_e_*, as shown in Figure 7.

As shown in Figure 7, n_0_ is the node base, n_l1_, n_l2_, and n_f_ are the number of nodes in the first layer, the second layer, and the full connection layer of the LSTM network, respectively, and the legend is n_0_Rn_l1_/n_l2_/n_f_. For example, 20R10/5/1 denotes n_0_ = 20, n_l1_ = 10 × n_0_ = 200, n_l2_ = 5 × n_0_ = 100, and n_f_ = n_0_ = 20. The neural networks of each structure show great differences in the early stage. Among them, the network with the 50R4/4/1 structure can achieve a higher accuracy faster, so the final network structure is n_l1_ = 200, n_l2_ = 200, and n_f_ = 50.

Learning rate is an essential parameter in the Adam optimizer, which determines whether and when the objective function converges to the minimum. Too large a learning rate may lead to the oscillation of the objective function, which is difficult to converge, and too small a learning rate will reduce the convergence speed. Therefore, choosing an appropriate learning rate is also a crucial link to improving the model’s accuracy. The learning rate is set to 0.0001, 0.00005, 0.00003, 0.00001, and 0.000005, and the corresponding results are shown in Table 2.

As shown in Table 2, when the learning rate is 0.00001, the model shows high prediction accuracy. When the learning rate increases or decreases, the model accuracy decreases, and the learning rate is determined as 0.00001.

#### 4.3.2. Test Results and Analysis

**Figure 8:**
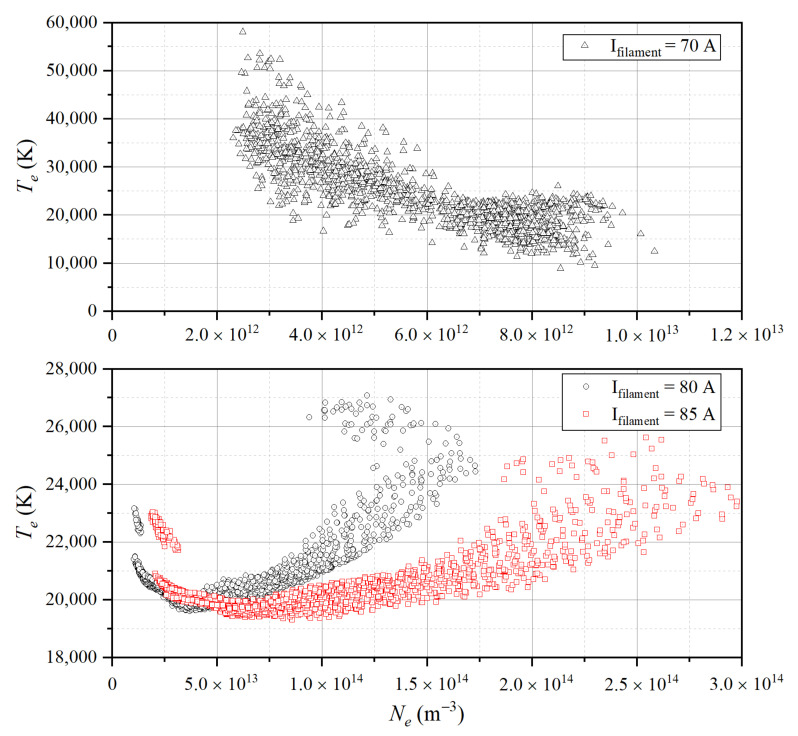
The electron density (*N_e_*) and electron temperature (*T_e_*) distribution of the data set.

The whole dataset includes 5186 groups of experimental data, in which *N_e_* ranges from 10^12^ to 10^14^ m^−3^ and *T_e_* ranges from 19,000 to 60,000 K. It is divided into 4150 training sets (including 1/4 verification sets) and 1036 test sets. The *N_e_* and *T_e_* distribution of the dataset are shown in Figure 8.

When the filament current (I_filament_) is 80 A and 85 A, the maximum *N_e_* is 1.75 × 10^14^ and 3.0 × 10^14^ m^−3^, respectively, and *T_e_* is mostly concentrated at 19,000 K–26,000 K. When the I_filament_ is 70 A, the data distribution is different from the other cases. There are two main reasons for this: first, in order to make the *T_e_* distribution range more extensive, we adjust the voltage of the accelerating grid when the I_filament_ is 70 A, so that the electrons emitted by the filament have a different kinetic energy and different *T_e_*. Compared with the case of higher plasma density, it is easier to adjust *T_e_* at low density. Another reason is that when the I_filament_ is 70 A, the collected I–V characteristic curve is difficult to reach saturation, so the results derived by traditional diagnosis methods are not accurate enough. This reason is discussed in detail in the prediction result analysis of *T_e_*.

**Figure 9:**
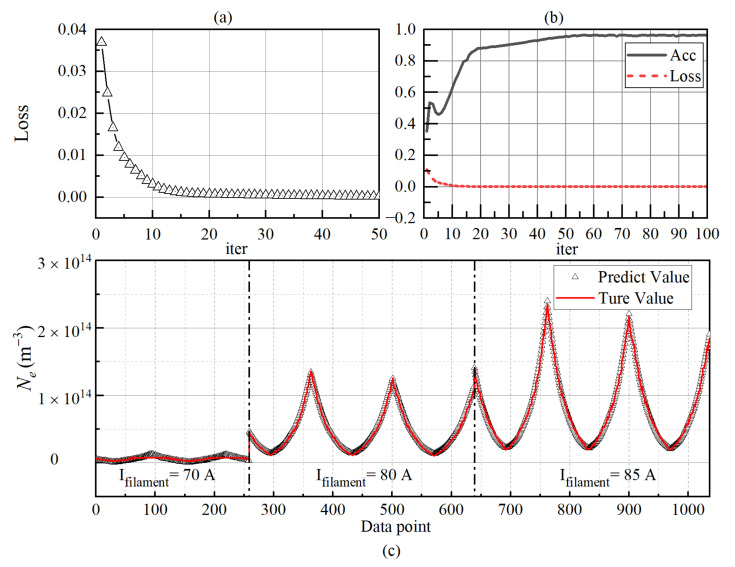
The training and prediction results of the *N_e_*. (**a**) The loss rate of the training set data; (**b**) The accuracy and loss of the verification set data; (**c**) Comparison of prediction results.

The test set is extracted proportionally from the I_filament_ at 70 A, 80 A, and 85 A. The training and prediction results of *N_e_* are shown in Figure 9.

The network learning situation for *N_e_* is shown in Figure 9a. With the increase in the number of iterations, the loss decreases rapidly and converges. Only about 50 iterations are needed, and the loss value decreases to the order of 10^−4^. At this time, the model achieves a good prediction effect. After each training iteration, the prediction ability of the verification set is tested. As shown in Figure 9b, the *Acc* has a local maximum before the tenth iteration and gradually rises and stabilizes after the tenth iteration. The networks reach the accuracy rate of 95% after 50 iterations. After completing the training and verification process of 100 iterations, 1036 groups of data from three groups are used to test the model, as shown in Figure 9c. Compared with the low-density plasma (10^12^ m^−3^), the network has more minor prediction errors for *N_e_* with large density. Of course, this is not entirely due to the network. Due to the weak current collected by the probe at low-density (I_filament_ = 70 A), the SNR of the collected signal is reduced, and it is easy for the I–V characteristic curve to be affected by the instrument, resulting in inaccurate collected data, and this error is irregular, which makes it difficult for the network to converge when using these data for training and to obtain accurate results when predicting. In addition, the data at low density are more vulnerable to sheath and edge charge effects.

**Figure 10:**
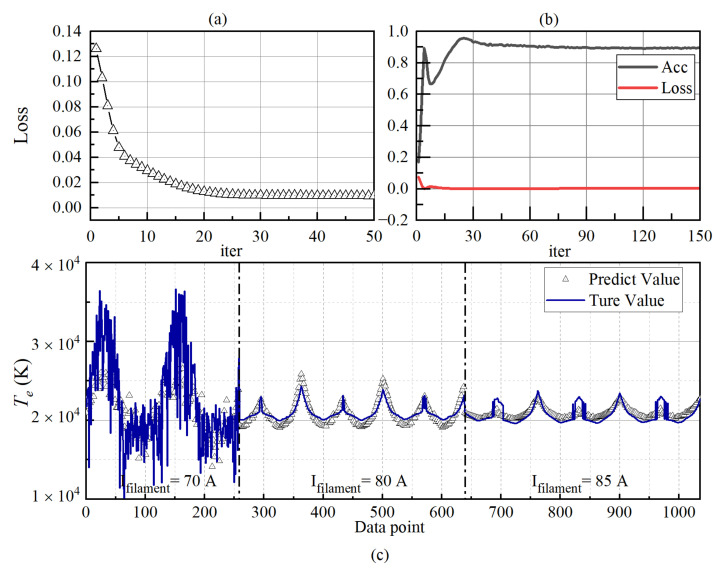
The training and prediction results of the *T_e_*. (**a**) The loss rate of the training set data; (**b**) The accuracy and loss rate of the verification set data; (**c**) Comparison of prediction results.

The training and prediction results of *T_e_* by the network are shown in Figure 10.

The training of *T_e_* in the LSTM network is more difficult than that of *N_e_*. At 50 iterations, the loss value reaches 0.01. After that, the loss decreases very slowly. After about 500 iterations, the loss can reach 0.005. For the verification set, such as the training process of *N_e_*, *Acc* reaches the maximum after experiencing a local maximum. At this time, the number of iterations is about 25. After that, *Acc* gradually decreases and stabilizes to 0.9 after about 200 iterations.

Figure 10c shows that the adverse effects of instrument acquisition accuracy, and the sheath and edge effect on the data are more significant at low density. However, the main reason for the poor accuracy of network prediction in the underdense plasma environment is that the calculation results of *T_e_* by traditional diagnostic methods are relatively inaccurate. In Section 2, there is a detailed *T_e_* calculation process in which obtaining *T_e_* requires the logarithmic fitting of the electron retardation region of the I–V characteristic curve. Theoretically, the relationship between the *I_e_* in the electron retardation region and the *V_B_* increases exponentially, and the logarithmic relationship is linear. However, in the underdense plasma, the electron retardation region of the I–V characteristic curve is relatively wide, and it is difficult to reach saturation. The I–V source data are more linear than the exponential function in the retardation region (as shown in Figure 3). The retardation curve presents a nonlinear shape (logarithmic function) after the logarithmic operation, so its slope changes significantly. Which section is selected for linear fitting has a large influence on *T_e_*, so the actual value curve of *T_e_* appears very unsmooth at low density. Theoretically, because the probe is continuously collected in space, the value of *T_e_* will not change suddenly, but the limitation of traditional diagnosis methods determines that the calculation result of *T_e_* has a significant error. Therefore, it is difficult to use such data to train and predict the network.

#### 4.3.3. Effect of Eliminating Contamination

**Figure 11:**
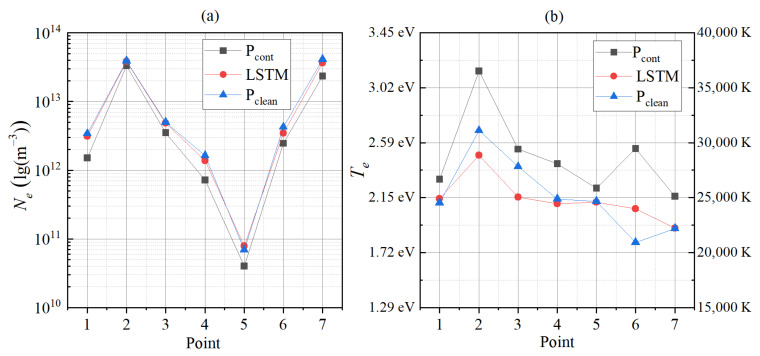
(**a**) The comparison results of the *N_e_*; (**b**) The comparison results of the *T_e_*.

We also test the prediction results of the network with the data collected by the contaminated probe (P_cont_) after the network training. A total of seven groups of I–V characteristic curves collected by P_cont_ and P_clean_ are used as raw data to diagnose the plasma by using traditional diagnosis methods and the LSTM network, respectively. The comparison results and errors of *N_e_* and *T_e_* are shown in Figure 11 and Table 3, respectively.

As shown in Figure 11a, for *N_e_*, the data collected by P_cont_ or P_clean_ are collected as the source data, and the results obtained by traditional diagnosis methods are significantly different. Table 3 shows that the absolute average error of seven groups of data reaches 40.33%. The reason is obvious. The absorption capacity of the probe with a contaminated layer for electrons is greatly weakened, resulting in the reduction in the collected electron saturation current and the calculated *N_e_*. The diagnosis results obtained using the LSTM network as the diagnosis method and the data collected by P_cont_ as the input are shown in the curve marked “LSTM” in Figure 11. It can be seen that using the machine learning method for plasma diagnosis can still obtain more accurate results even when there is a certain contaminated layer on the probe surface. The average absolute error of *N_e_* obtained by the LSTM network is reduced from 40.33% to 10.69%. In other words, the LSTM plasma diagnosis network has the effect of partially compensating for the contamination on the probe surface.

Figure 11b shows the diagnostic results of *T_e_*. Similar to *N_e_*, the LSTM network has a certain compensation effect for the probe surface’s contamination; it can reduce the absolute average error from 14.81% to 5.05%. Unlike *N_e_*, the data collected by P_cont_ are used as the source data, and the results derived by traditional diagnostic methods are relatively large compared with the actual value because the contaminated layer may change the work function of the material of the probe itself. In addition, when using the traditional method for plasma diagnosis, we first calculate *T_e_* and then combine the electron saturation current to derive *N_e_*. From Equation (7), it can be seen that the small *N_e_* is also partly due to the sizeable diagnostic result of *T_e_*. Compared with *N_e_*, the consistency of LSTM’s results of *T_e_* is low. For example, in groups 1, 4, and 7, the LSTM network diagnosis results are close to the actual value, but in group 6, although the error caused by the contaminated layer can be partially compensated, there is still a 14.54% error with the actual value. The third group of data is unusual. The LSTM prediction results do not only reduce the error, but also increase the error from 5.70% to −9.98%. This problem seems to need more *T_e_* data with a larger distribution range to train the network.

Another advantage of plasma diagnosis using the LSTM network is that there is no coupling process in the diagnosis process of *N_e_* and *T_e_*; that is, their acquisition is relatively independent. In this way, it avoids the large influence on the result of *N_e_* due to the significant error of *T_e_*, which is common in traditional diagnosis methods.

## 5. Conclusions

In this paper, we use the machine learning method to train an LSTM network based on Langmuir probe data to diagnose plasma. This network is separated from the gas discharge device and has more robust applicability. Compared with the traditional diagnosis method based on OML theory, the LSTM network only needs to input 1/10 data points, which significantly reduces the error caused by “human factors” in the traditional diagnosis method and has a certain compensation effect on the contamination of the probe surface. The training and test data of the network are from the experimental data of the plasma vacuum chamber. On the one hand, through the coverage experiment of an extensive density range, it is proved that after about 50 to 200 iterations, the network can obtain more than 95% prediction accuracy of *N_e_* and more than 90% prediction accuracy of *T_e_* respectively. On the other hand, through the contrast experiments designed separately, compared with the traditional diagnosis method, the LSTM network can reduce the electron density error caused by contamination from 40.33% to 10.69%, and the electron temperature error from 14.81% to 5.05%. That is, the LSTM network can obtain relatively accurate results even using the data obtained by the probe whose surface is partially contaminated.

This network is lightweight and requires less input data than traditional diagnosis methods do. In the future, it can be applied to ionospheric plasma diagnosis by carrying satellites, which can greatly save downlink data and improve the spatial resolution of ionospheric detection.

Although there is little research on the application of the machine learning method to plasma diagnosis, machine learning is a very flexible tool and is suitable for the diagnosis of various plasmas. In our next research, we will further optimize the LSTM network proposed in this paper to improve its prediction accuracy, especially for *T_e_*. In addition, we are also ready to apply the machine learning method to other plasmas, such as magnetron plasma or ionospheric plasma.

## Figures and Tables

**Table 1:** The comparison of plasma parameters from P_clean_ and P_cont_.

	V_p_	I_e0_	N_e_	T_e_
P_clean_-Upward	1.0 V	9.8014 × 10^−7^ A	2.1040 × 10^12^ m^−3^	0.7794 eV
P_clean_-Downward	1.0 V	9.6144 × 10^−7^ A	2.0742 × 10^12^ m^−3^	0.7717 eV
P_cont_-Upward	0 V	1.6280 × 10^−7^ A	5.1256 × 10^11^ m^−3^	0.3623 eV
P_cont_-Downward	3.2 V	2.4366 × 10^−7^ A	5.7090 × 10^11^ m^−3^	0.6543 eV

**Table 2:** The influence of different learning rates on the model.

η	0.0001	0.00005	0.00003	0.00001	0.000005
RMSE	0.00507	0.00511	0.00511	0.00582	0.00610
MAPE	25.61891	23.37818	11.50008	10.64523	13.30315

**Table 3:** Comparison of errors in predicting electron density (*N_e_*) and electron temperature (*T_e_*) using traditional diagnostic methods and LSTM.

		1	2	3	4	5	6	7	|Mean|
*N_e_*	Traditional	−55.81%	−14.28%	−29.53%	−56.03%	−41.95%	−41.98%	−42.75%	40.33%
	LSTM	−8.67%	−2.23%	−3.42%	−15.96%	14.46%	−18.54%	−11.57%	10.69%
*T_e_*	Traditional	8.78%	17.43%	5.70%	12.92%	4.87%	40.77%	13.20%	14.81%
	LSTM	1.46%	−7.20%	−9.98%	−1.80%	−0.27%	14.54%	0.11%	5.05%

## Data Availability

The data presented in this study are available on request from the corresponding author upon reasonable request.
